# Patient and clinician preferences for diabetes management among older adults with co-morbid HIV: A qualitative exploration

**DOI:** 10.1371/journal.pone.0303499

**Published:** 2024-05-14

**Authors:** Allison P. Pack, Mary Clare Masters, Rachel O’Conor, Kenya Alcantara, Sophia Svoboda, Reneaki Smith, Fangyu Yeh, Guisselle Wismer, Amisha Wallia, Stacy C. Bailey

**Affiliations:** 1 Division of General Internal Medicine, Feinberg School of Medicine, Northwestern University, Chicago, Illinois, United States of America; 2 Department of Medicine, Feinberg School of Medicine, Northwestern University, Chicago, Illinois, United States of America; 3 Institute for Public Health and Medicine, Feinberg School of Medicine, Northwestern University, Chicago, Illinois, United States of America; University of Benghazi, LIBYA

## Abstract

**Background:**

Older adults with HIV are at increased risk of developing certain chronic health conditions including type 2 diabetes mellitus (T2DM). As the number and complexity of conditions increases, so do treatment and health care needs. We explored patient and clinician preferences for HIV+T2DM care and perceived solutions to improving care.

**Methods:**

We conducted an exploratory qualitative study comprised of individual in-depth interviews. Participants included English-speaking patients aged 50 and older living with HIV and T2DM and infectious disease (ID) and primary care (PC) clinicians from a large academic health center in Chicago. Thematic analysis drew from the Framework Method.

**Results:**

A total of 19 patient and 10 clinician participants were interviewed. Many patients reported seeking HIV and T2DM care from the same clinician; they valued rapport and a ‘one-stop-shop’. Others reported having separate clinicians; they valued perceived expertise and specialty care. Nearly all clinicians reported comfort screening for T2DM and initiating first line oral therapy; ID clinicians reported placing referrals for newer, complex therapies. Patients would like educational support for T2DM management; clinicians would like to learn more about newer therapies and easier referral processes.

**Conclusions:**

Patient-centered care includes managing T2DM from a variety of clinical settings for individuals with HIV, yet strategies are needed to better support clinicians. Future research should examine how best to implement these strategies.

## Introduction

Adults with HIV are living longer lives and are now more likely to experience comorbid conditions that commonly occur with age, such as type 2 diabetes mellitus (T2DM) [1, 2]. Prior research indicates adults with HIV are up to four times as likely to develop T2DM than those without HIV, due in part to metabolic factors related to HIV and to certain HIV therapies [2]. Those with HIV+T2DM are also more likely to experience T2DM-related complications and adverse health outcomes [3–5]. Current estimates from the United States reveal the prevalence of T2DM is greater than 18% among those also living with HIV, compared to less than 12% of the general population [6, 7].

T2DM is usually managed by primary care (PC) clinicians [8]. However, because of the stigmatized nature of HIV and a system of care that has historically siloed the condition, many older adults with HIV view infectious disease (ID) clinicians as their primary source of healthcare, relying on them for non-infectious chronic disease management as well as HIV care [9]. Yet, the shrinking number of HIV care clinicians [10], coupled with the complex treatment and health needs of individuals with HIV and multiple other chronic conditions [11–13] is creating a shift in HIV care. Individuals with HIV and T2DM are increasingly seeking care from primary care (PC) clinicians or endocrinologists [10]. In this transitional period, it is useful to understand patient and clinician preferences for care as well as perceived solutions to care improvement. This is particularly the case for patients struggling to effectively manage their conditions, yet few studies have sought to examine this; those that have, have largely focused on self-management strategies as opposed to care delivery [14–17].

To fill this research gap, we conducted an exploratory, qualitative study among patients with HIV+T2DM and clinicians. Among patient participants, we explored preferences for T2DM care; emphasis was given to understanding whether there was a particular type of clinician from whom these individuals prefer to seek care and whether they would be receptive to receiving additional educational support for T2DM management, beyond the point of care. Similarly, among clinician participants, we sought to identify current practices for T2DM screening and management, and to solicit ideas for enhanced management strategies.

## Methods

We conducted exploratory in-depth qualitative interviews among patients and clinicians from one large academic health center in Chicago. Demographic data from patient participants has been previously published in a separate study examining medication non-adherence [18]. The Northwestern University Institutional Review Board approved all research activities (SP0076311).

### Eligibility criteria

Patients were eligible to participate in the study if they were: 1) English-speaking, 2) aged 50 or older; 3) living with co-morbid HIV+T2DM, 4) taking oral medications to treat their HIV and diabetes, and 5) at risk of potential medication non-adherence according to clinical values obtained in the previous year (high viral load (HIV RNA >50 copies/ml) and/or HbA1C ≥7.5%). Patients were excluded if they had any vision, hearing, or cognition challenges that would inhibit informed consent.

Eligibility criteria for clinicians included employment by the participating health center within any infectious disease, primary care, or endocrinology practice. To be eligible, clinician participants also had to self-report experience managing HIV+T2DM among older adults.

### Participant recruitment

Potentially eligible patient participants were identified via an electronic health record (EHR) query. Trained research coordinators (RC) contacted participants by phone, confirmed eligibility, and engaged participants in an electronic consent process before conducting the interview.

Potentially eligible clinician participants were identified via professional networks and snowball sampling. Specifically, we leveraged existing clinician networks, including practice administrators and clinicians in PC, ID, or endocrinology. Initially, clinicians who were believed to meet eligibility criteria were emailed information about the study and asked to contact study staff if they were interested in participating; they were then encouraged to forward the email to others. Those who contacted the study staff and confirmed eligibility were sent an electronic consent form they signed prior to their interview.

We sought to recruit enough patient and clinician participants to achieve thematic saturation [19, 20].

### Data collection

Interviews with patient participants from the participating academic health center took place between October 2022 and January 2023. Each interview lasted approximately one hour and was conducted by a trained RC. At the end of the interview, the RC administered a brief sociodemographic questionnaire and validated measures of patient activation and health literacy. Patient activation was assessed using the Consumer Health Activation Index [21] and health literacy was assessed using a three-item screener [22]. These measures were included to further characterize the patient participant sample in terms of how well they are able to understand and use health information, and how activated they are in their own healthcare.

Interviews with clinician participants took place between March 2023 and July 2023. These lasted approximately 45 minutes and were conducted by a PhD-level qualitative researcher. Clinician participants completed a brief demographic questionnaire asynchronously.

Interview guides included exploratory questions designed to solicit an understanding of participant preferences and current experiences (see Supplementary Materials). All interviews were conducted virtually over web-based conferencing software and were audio recorded, transcribed, and supplemented by detailed field notes. Quantitative data was captured in REDCap, and all participants were compensated for their time.

### Analysis

We drew from the Framework Method, an inductive and deductive approach to thematic analysis of qualitative data [23]. To begin, we familiarized ourselves with content by reading transcripts and writing additional memos. Information gained during this process was used to develop two codebooks–one for the patient transcripts and a separate one for the physicians [24]. *A priori* codes were drawn from the interview guides, while *emergent* codes were drawn from the transcripts and memos. We piloted the codebooks on transcripts from four patient and four clinician participants using NVivo qualitative software. These initial transcripts were double coded with all coding reconciled. Remaining transcripts were independently coded; however, regularly held meetings offered an opportunity to review coding and discuss emergent themes [25]. For our data reduction step, we then created Excel matrices with rows representing individual participants and columns containing information pertinent to individual codes; illustrative quotes were also included [25]. We identified the themes presented below by summarizing the coding content across participants [23, 25]. To analyze the quantitative data, we conducted descriptive statistics using SAS 9.4 software.

## Results

### Patient participant characteristics

The characteristics of patient participants are reported in Table 1. They included 19 individuals whose average age was 61. The majority were male (90%). Most self-identified as being White (53%), non-Hispanic (84%), and lesbian or gay (83%). The majority had a bachelor’s degree or higher (63%), and nearly half were working for pay (41%). Just over a quarter reported their HIV and diabetes were both managed by a primary care provider (n = 5, data not shown). A sizable proportion of participants was classified as having low health activation (47%), and most were classified as having adequate health literacy (90%).

**Table 1:** Patient participant characteristics (n = 19).

**Age**	
Mean (SD)	61.3 (6)
Median (Range)	61 (51–72)
**Gender Identity, n (%)**	
Male	17 (90)
Female	2 (10)
**Sex Orientation, n (%)**	
Straight or Heterosexual	3 (17)
Lesbian or Gay	15 (83)
**Ethnicity, n (%)**	
Hispanic	3 (16)
Not Hispanic	16 (84)
**Race, n (%)**	
White/Caucasian	10 (53)
Black/African American	5 (26)
Asian	1 (5)
Hispanic/Latino/Spanish	1 (5)
Other	2 (10)
**Education, n (%)**	
High School	2 (10)
Some College	5 (26)
Bachelors Degree	6 (32)
Masters Degree	4 (21)
Doctorate	2 (10)
**Employment Status, n (%)**	
Working for pay	8 (42)
Unemployed	1 (5)
Unable to work due to disability or illness	3 (16)
Retired	5 (26)
Mixed	2 (10)
**High VL, n (%)**	6 (32)
**High HbA1C, n (%)**	16 (84)
**CHEW Health Literacy, n (%)**	
Limited	8 (42)
Adequate	11 (58)
**Patient Activation Level (CHAI), n (%)**	
Low	9 (47)
Moderate	8 (42)
High	2 (10)

### Clinician participant characteristics

Characteristics of clinician participants are presented in Table 2. Participants included 8 clinicians from infectious disease and 2 from primary care; 1 clinician had a dual appointment in infectious disease and primary care. The majority were male (60%) and most self-identified as being White (80%). Years in practice varied, ranging from less than five years to 20 or more.

**Table 2:** Clinician characteristics (n = 10).

**Clinician Type**	**n (%)**
Infectious Disease	8 (80)
Primary Care	2 (20)
**Years Been in Practice**	**n (%)**
< 5 years	2 (20)
5–9 years	2 (20)
10–14 years	2 (20)
15–19 years	1 (10)
20+ years	3 (30)
**Gender Identity**	**n (%)**
Male	6 (60)
Female	4 (40)
**Ethnicity**	**n (%)**
Hispanic	-
Not Hispanic	10 (100)
**Race**	**n (%)**
White/Caucasian	8 (80)
Asian	2 (20)

### Thematic findings

Thematic findings, identified using the Framework Method, are presented below. We first present themes pertaining to the topic of ‘preferences and experiences with care’ and subsequently present those pertaining to ‘solutions for improving care’.

### Preferences and experiences with care

#### Theme 1: Patient participants reported varying preferences for HIV and T2DM care

Approximately half the sample reported a preference for having the same clinician manage their HIV and T2DM. Of these, only a few noted their ID clinician manages both conditions, while the rest indicated their conditions are managed by their PC clinician. Most often, participants explained their preference to stay with the same clinician by claiming they found it convenient to have both conditions addressed in a single visit (thus reducing the need for additional appointments), or they had established rapport with that clinician.

For example, one participant who prefers to stay with his ID clinician explained:

*“It’s less appointments*, *[chuckles] that’s one*. *Two*, *I prefer the one-stop shop method here*. *We talk about everything when I go to the doctor*. *He’s looking at all the numbers at one time and I don’t have to go to somebody else*. *[That] makes it easy for me*.*”*

*(patient identification number (PID) 7*, *64-year-old Black male)*

Another participant who reported preferring to stay with his PC clinician, explained his preference by describing the rapport he’s built over the years:

*“He’s known me for a long time so just the fact that I’ve been going to see him since 2009*. *We’ve built a rapport*, *and he covers me*. *He takes a total blood panel every time I see him*. *He keeps in touch with me*, *and I can communicate with him online and get responses*.*”*

*(PID 29*, *72-year-old White male)*

Finally, separate from the convenience of a ‘one-stop shop’ and good rapport, one participant perceived having a single clinician manage his HIV and T2DM ensures that clinician has all the information needed to inform health care decisions. He worried having multiple clinicians could complicate the exchange of pertinent information:

*“The only thing I worry about is the ability for communication between providers to be good…”*

*(PID 27*, *61-year-old White male)*

Nevertheless, nearly half of all patient participants reported having separate clinicians for HIV and T2DM management. Of these, most indicated the arrangement was the result of a referral, either from ID to PC or from PC to endocrinology. Yet, it was something they appreciated. For example, one participant described how he perceived that having separate clinicians allowed his ID clinician to more effectively do “his job”:

*“… Initially*, *when I first started with my current HIV doctor*, *he would address other issues too*, *and then he scaled it back to where he only handled the HIV and made me see my primary care for all other issues–which is his job*, *basically*. *He’s an infectious disease doctor*, *so he’s there to address those*, *not everything else under the sun… A one-stop-shop would be nice*, *but at the same time*, *I think two is more beneficial*.*”*

*(PID 17*, *51-year-old White male)*

Another similarly explained a referral from PC to an endocrinologist was beneficial as they are “the people who deal with diabetes”:

*“I see an endocrinologist for the diabetes… Because my doctor for HIV doesn’t know much about diabetes*. *He tells me you have to see an endocrinologist*: *‘You need to make an appointment in endocrinology because they’re the people who deal with diabetes*.*’… I basically go to the person who knows what they’re talking about… I don’t think that there are providers who do both*.*”*

*(PID 32*, *69-year-old White male)*

Others perceived that endocrinologists could work with their PC clinicians by offering insight into “new drugs that are coming out” (PID 26, 59-year-old White male) or by adjusting more complex therapies for T2DM (PID 1, 67-year-old Asian male).

There were, however, a few participants who suggested they prefer separate clinicians manage their conditions–regardless of whether they had received a referral. One participant explained she prefers an endocrinologist manage her T2DM “because they specialize in that particular area, and I feel more comfortable with that” (PID 33, 71-year-old Black female) and another noted he likes having his ID and PC clinicians work together to manage his health (PID 15, 66-year-old Hispanic male).

#### Theme 2: Clinicians reported regularly screening patients for T2DM

All clinician participants indicated they screen patients at least annually for diabetes using hemoglobin A1C (HbA1C) or fasting blood glucose tests; typically, these tests are included in a larger panel of laboratory tests that are part of routine HIV care [26]. A majority of participants noted they also assess a patient’s risk of diabetes through other methods as well, including urinalyses, and patient characteristics such as age, weight, race, ethnicity, genetic predisposition, and/or patient reported symptoms. For patients with numerous risk factors, clinician participants noted they may conduct screening more frequently. One participant explained:

*“The screening I usually order is a hemoglobin A1C*. *It’s at least annually*. *[F]or some patients at high risk who*, *say*, *maybe just have either pre-diabetes or other chronic cardiovascular conditions*, *it would probably be every six months… As part of routine care*, *we ask about symptoms*.*”*

*(PID 3*, *female ID physician with 20 or more years in practice)*

Only a couple of clinician participants mentioned a need to examine how long patients have been living with HIV and whether they have been on antiretroviral medication that could elevate their risk of diabetes, though in general, screening practices for T2DM did not differ by a patient’s HIV serostatus.

*“I don’t have a different approach for that population specifically… The screening guidelines that are out there from USPSTF and from ADA and from other groups like American Association of Clinical Endocrinologists*, *they don’t really mention anything about HIV status as a risk factor*. *[Though] I know some of the older HIV drugs and the protease inhibitors caused lipid abnormalities… that might be related to diabetes risk*.*”*

*(PID 6*, *male PC physician with 15 to 19 years in practice)*

All clinician participants noted they were comfortable screening patients for diabetes, as laboratory assessments are a common component of HIV and/or general wellness care.

#### Theme 3: Most clinician participants reported comfort initiating first line oral therapy, though preferences were noted for primary care and endocrinology to manage more complicated cases

Most ID clinician participants noted comfort diagnosing T2DM and starting patients on first line oral therapy. However, they often reported referring patients with uncontrolled HbA1C levels to primary care or endocrinology; those departments were perceived to be more adept at prescribing complex therapies, insulin, and/or additional support services such as diabetes educators and nutritionists. One clinician participant explained:

*“If [the patient is] not getting anywhere with [lifestyle modifications]*, *then I’d be talking to them about starting first line therapy*, *which generally is Metformin for most of the patients*. *I’m pretty comfortable prescribing first-line therapy*. *If … the frontline therapy doesn’t seem to be helping much*, *that’s when I’m generally referring to endocrinology or primary care*.*”*

*(PID 1*, *male ID physician with 10 to 14 years in experience)*

A few others described how multimorbidity factors into their decisions to refer a patient to primary care or endocrinology. For example:

*I’ll engage primary care if the numbers of medical comorbidities that the patient has become too difficult for me to manage*. *I am not just an HIV provider*. *I see many other ID patients*. *When there’s a lot of complexity to the non-HIV related medical problems*, *it becomes unwieldy for me to manage*. *If it’s just diabetes*, *and managing all the effects around diabetes*, *I’m okay with that and we’ll engage endocrinology*. *When there are additive medical problems*, *that’s when I engage primary care*.*”*

*(PID 5*, *male ID physician with 10 to 14 years in practice)*

In addition to the challenges of managing multiple chronic conditions, there was a broad recognition among most ID participants that staying current on rapidly changing diabetes medications was beyond their capacity, given their existing clinic resources, time, and competing priorities. Nevertheless, some suggested early career clinicians may be more comfortable with T2DM management as it comprises part of their training, while another participant suggested that clinicians who have long been a medical ‘home’ for patients with HIV may also feel more comfortable managing T2DM when he noted:

*“A lot of [ID] physicians in my division*, *the older physicians will self-manage all the conditions–and that’s noble–but I feel like a lot of times I’m just not up to date on the general medicine stuff that I should be*, *especially for diabetes*. *Everyone who needs medicine*, *I’m outsourcing to a primary care doctor or endocrinologist*.*”*

*(PID 9*, *male ID physician with 5 to 9 years in practice)*

Primary care clinicians in our sample, on the other hand, were reportedly comfortable starting and managing patients on a wide range of T2DM therapies and they were open to receiving referrals from ID.

### Solutions for improving care

#### Theme 1: Patient participants were receptive to the idea of utilizing the patient portal to further support the management of T2DM

Beyond support from clinicians, when patient participants were asked what they thought of educational interventions to enhance T2DM management, most suggested they would be open to receiving information via the patient portal (i.e., MyChart); some were receptive to text messages. This included information supporting healthy lifestyle choices, such as diabetes-friendly recipes and exercise ideas. One participant explained he’d like to receive:

*“Recipes or something that would be good to refer to… I’m always interested where on the glycemic index do certain foods*, *where do they fall*? *Are they high*, *medium*, *low*, *or whatever*. *If that was a resource on there*, *that would be something that would be useful*. *Maybe if they had any links to exercise programs …”*

*(PID 16*, *69-year-old White male)*

A few others noted they would prefer information describing how to access support from others, including diabetes educators, dieticians, and/or stories from other people with HIV+T2DM:

*“I would even like hearing stories of other people*, *their experiences… Because you sometimes do think that you’re the only one struggling with this and you’re not*. *You’re finding out that actually this is a very common thing*, *and it happens so often*.*”*

*(PID 30*, *54-year-old White male)*

One participant perceived the patient portal could be useful for delivering newly published T2DM research. She suggested “some people are stuck in the old ways” (PID 12, 65-year-old Black female) and are unaware of the rapidly changing treatment landscape. She felt information posted within the portal could help her to stay abreast of new treatment options.

#### Theme 2: Clinician participants also suggested a variety of ways education and additional support staff could potentially enhance care

Although some participants indicated an ideal world comprise a clear “division of labor where you have a primary care doctor who’s taking care of most things and the ID doctor taking care of the HIV” (PID 6, male PC physician with 15 to 19 years in practice), there was also recognition that “we have to think of things [as being] patient-centered and think about what they want and what their needs are” (PID 1, male ID physician with 10 to 14 years in practice). As such, several ID participants suggested they could benefit from additional education or support staff (i.e., diabetes educators, case managers, and pharmacists). Education could be delivered as quality improvement, tailored to ID clinicians to expand comfort with new T2DM therapies. One ID clinician suggested such education could be delivered in the form of cross-division seminars simultaneously designed to increase familiarity and rapport among physicians. Support staff could further enhance clinician education while also offering additional services–and education–to patients with T2DM. For example, a diabetes educator was mentioned by some participants: "That’d be amazing to have a diabetes educator or any type of patient educator about lifestyle modifications and other things like your glucometer. Their glucometers always break and don’t work. Just troubleshooting that kind of stuff for patients would be better. That would be amazing.” (PID3, female ID clinician with 20+ years of experience).

Detailed T2DM educational materials for patients were also suggested for enhancing management and care. Some clinician participants acknowledged that while this information is often readily available in primary care and endocrinology, brief and patient-friendly education should also be integrated into ID practices.

#### Theme 3: Clinician participants perceived enhancements to the EHR could improve care

In addition to educational supports, clinician participants also suggested EHR enhancements could improve care. In fact, these suggestions were the most frequent type of suggested provided by clinician participants; they included optimizing the referral process, automating screening reminders, and facilitating communication between physicians.

ID clinicians reported wanting a feature embedded within the EHR to help them appropriately select clinicians for referral purposes. Many indicated that at a large, academic medical center they lack “familiarity with who’s on the other side of the referral” and they would like to “build up a rapport with that other person” (PID 10, female ID physician with 5 to 9 years in practice). Another participant explained:

*“I think it could be helpful having someone engaged with us who really wants to work with our HIV population… To be honest*, *internal medicine providers that do ‘day-in and day-out’ primary care are a little better at it*. *[Knowing] people we can send our patients to*, *is always helpful to us and would really serve our patient population*.*"*

*(PID 1*, *male ID physician with 10 to 14 years in practice)*

Others noted they would like automated screening reminders embedded within the EHR system for tests other than the HbA1C and for communication to indicate “ownership” of activities, particularly when other clinicians are involved. For example,

*“Who’s screening their eyes? Have they gotten in to see ophthalmology? Who’s checked their proteinuria to make sure their kidneys are okay? That’s sometimes where it gets a little muddy… I tend to assume the PCP and the endocrinologist are managing all the diabetes-related complications and screenings.”*

*(PID 3*, *female ID physician with 20 or more years in practice)*

Communication suggestions also included an automated feature to prompt brief messages between referring and receiving clinicians. Unlike notes in a patient’s medical record, which are perceived to be “generally pretty poor” (PID 6, male PC physician with 15 to 19 years in practice), these messages would focus on why a referral was placed and what follow up might be needed, especially since referrals take time. One participant further described this when he said:

*“It’s always nice just to have a quick message saying*, *‘I saw your patient*, *and here’s what we’re doing with them*.*’ Nice to get that feedback and to know what’s going on exactly…*. *I feel like I don’t get as much good feedback from the [other departments] when I have someone there*. *I think they assume I’m going to look at the notes and rely on that*, *which is okay*.*”*

*(PID 1*, *male ID physician with 10 to 14 years in practice)*

## Discussion

In this qualitative study, we sought to better understand patient and clinician preferences for the management of T2DM among older adults with HIV. Analysis identified several key themes.

First, we noted the importance of providing T2DM care in a variety of clinical settings, with some patient participants reporting a preference to receive HIV and T2DM care from the same clinician, and others preferring separate clinicians. While research is examining how best to integrate services [27], understanding that some patients may prefer to see separate clinicians is also important. Honoring patient preference is associated with improved care engagement and health outcomes, as it is a key component of patient-centered care [28]. However, this becomes increasingly difficult if the number of ID clinicians offering HIV care continues to dwindle [10].

We additionally found that infectious disease clinician participants largely reported referring patients to primary care or endocrinology when T2DM management requires newer or more complex therapies. Nevertheless, they also noted a smoother referral process is needed. Focused or relational referrals, in which physicians have a clear understanding of the individual to whom they are referring–as opposed to just the department–can build relationships between physicians and lead to better health system and patient outcomes [29]. Other studies conducted among individuals with HIV have similarly noted a need for more effective referral processes for diabetes care [30]. This is critical to address as the number of individuals with HIV+T2DM is expected to increase [6]. Similarly, we found clinician participants wanted increased transparency in “ownership” of clinical activities when care is shared between multiple clinicians. Analogous results have been identified by another study conducted among multiple Department of Veterans Affairs Medical Centers; the authors of that study found that for team-based care to be effective, there must be coordination of services [17].

Finally, patient and clinician participants in our study expressed an openness and/or interest in a variety of educational and EHR-based strategies to enhance T2DM care among individuals with HIV. Patient participants, the majority of whom were classified as having adequate health literacy, noted they are open to receiving T2DM management support via the patient portal, and clinicians supported the idea. Although current literature reveals portal usage does not necessarily result in improved clinical outcomes for diabetes, the potential exists [31]; portal usage has also been associated with increased patient activation [32]. Future research should explore strategies for providing self-management strategies via the patient portal. Furthermore, our study like others, suggests ID physicians with less than five years of experience, and/or those who have long been the medical ‘home’ for individuals with HIV, are more likely to provide primary care services [9]. To better accommodate patients who prefer to receive T2DM care from their ID physicians, participants suggested it may be useful to ensure more ID physicians are knowledgeable about a select number of newer T2DM therapies through educational opportunities. Whether and how to impart this knowledge, however, will need to be examined in future research.

This study is not without limitations. First, we interviewed patients and clinicians from a single academic health center; given regional variation in approaches to HIV care, our results may differ from those gathered in other locations. The number of clinicians we were able to interview across disciplines at this one health center was low; nevertheless, their opinions are still valuable to understanding care preferences and perceived solutions [33] and the total sample size is considered sufficient for thematic saturation [19, 20]. One reason for the low number may be that we required clinician participants to report having experience providing care to individuals with HIV+T2DM. Endocrinologists who responded to our snowball recruitment emails, all reported that they do not perceive themselves as having patients with HIV, rendering them ineligible, despite nearly one third of patient participants in our sample reported seeing an endocrinologist for their T2DM. Given the current shortage of endocrinologists, those eligible may not have responded because they did not have the time [34, 35].

## Conclusions

Findings from this study highlight differing preferences regarding T2DM management among patient participants. Results also suggest enhanced referral processes, as well as the delivery of T2DM education for patients and ID clinicians could improve T2DM management among individuals with HIV. Future research should examine whether and how to implement these strategies between infectious disease, primary care, and endocrinology. Lessons learned could be applied to consultation models in other disease contexts.

## Supporting information

S1 Checklist Human participants research checklist. (DOCX)
